# Impact of Delayed Diagnosis in IBD on Clinical Outcomes and Healthcare Delivery

**DOI:** 10.3390/diagnostics16071043

**Published:** 2026-03-30

**Authors:** Uday N. Shivaji, Snehali Majumder, Abhishek Rao, Alina Bazarova, Tommaso L. Parigi, Subrata Ghosh, Marietta Iacucci

**Affiliations:** 1 Department of Gastroenterology, Institute of Immunology and Immunotherapy, University of Birmingham, Birmingham B15 2TT, UK; udaynagesh.shivaji.dr@narayanahealth.org (U.N.S.); snehali.majumder@narayanahealth.org (S.M.);; 2 Department of Gastroenterology, Mazumdar Shaw Medical Centre, Narayana Health City, Bangalore 560099, India; 3 Department of Gastroenterology, University Hospitals Birmingham, NHS Foundation Trust, Birmingham B15 2GW, UK; abhishek.rao@uhb.nhs.uk; 4 National Institute for Health Research (NIHR), Birmingham Biomedical Research Centre, University Hospitals Birmingham, BirminghamB15 2GW, UK; 5 Department of Clinical Research, Narayana Health City, Bangalore 560099, India; 6 APC Microbiome Ireland, College of Medicine and Health, University College Cork, T12 K8AF Cork, Ireland; 7 Biological Physics, Institute for Biological Physics, University of Cologne, 50923 Cologne, Germany

**Keywords:** IBD, delayed diagnosis of IBD, sustained chronic inflammation, complications of delayed diagnosis, challenges in endoscopy services

## Abstract

**Background:** Delays in diagnosis are unfortunately quite common in most health systems. It is apparent that timely diagnosis is more likely to have a favourable outcome. However, there may be many reasons why timely diagnosis is not always achieved. The aim of our study was to report on the impact of delays on IBD-related adverse outcomes (AOs). **Methods:** New patients referred for suspected IBD to a single tertiary care centre between January 2013 to December 2017 were identified using EMR. For purposes of the study, a cut-off time was set by investigators for each delay-type based on best average hospital waiting times. The reasons for delays in patient journey until start of treatment and data on pre-defined AOs (steroid & other rescue therapies, hospitalisations, surgery) were recorded for each patient until end of June 2021. The data were analysed using multiple Pearson correlations and Cox proportional Hazard model to determine whether there is a difference in survival without AOs between patients with and without a delay. **Results:** Total of 105 patients were identified using stringent criteria (M = 58; median age = 32 y) with a long median follow-up of 55 months. 65, 27 and 13 patients had final diagnosis of Ulcerative colitis, Crohn’s disease and Unclassified colitis respectively, and analysed collectively. In our cohort, the longest delay-types noted were—patients seeking medical attention (median = 4 months; range 1 to 84 months), arranging gastroenterology clinic review after referral from primary care (median = 5 weeks; range 1 to 30 weeks), and waiting for index endoscopy (median = 3 weeks; 1 to 36 weeks). Patient stratification based on delay-type using specific cut-off times for each showed a statistically significant difference in survival without AOs for all (when comparing delay v/s no delay). **Conclusions:** In our cohort we report that delays, and subsequent untreated chronic inflammation, leads to poor outcomes in patients with newly diagnosed IBD regardless of whether delays are patient-related or health-system-related. Also, cumulative delays in the hospital appear to increase the use of biologics in consecutive years. Understanding these factors help rectify and offer long-term solutions.

## 1. Background

Inflammatory bowel diseases (IBDs) can cause significant physical and psychological distress, leading to a diminished quality of life for those affected. The delayed diagnosis of IBD is not uncommon, and it can have profound consequences on patients’ well-being and long-term prognosis [1].

**Figure 1:**
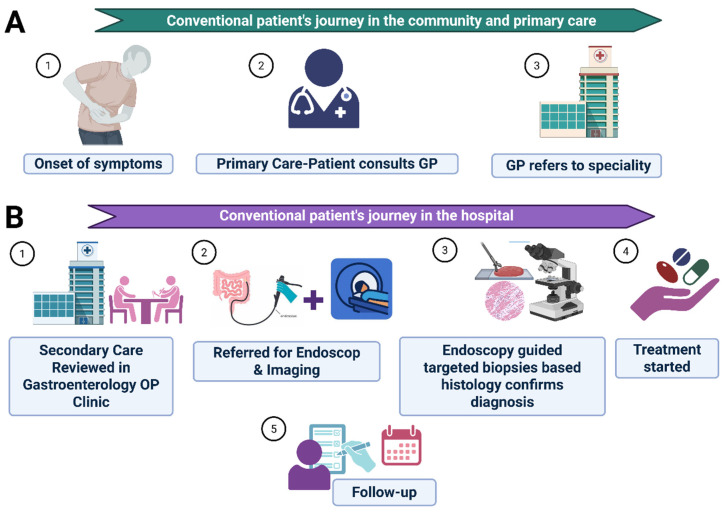
Typical patient journey from symptom onset to follow-up in gastrointestinal disease. (**A**) Community and primary care pathway: the patient develops symptoms (1), consults a general practitioner (GP) (2), and is referred to secondary care (3). (**B**) Hospital pathway: The patient is reviewed in gastroenterology outpatient clinic (1), referred for endoscopy and imaging (2), and undergoes endoscopy-guided biopsies for histological confirmation of diagnosis (3). Treatment is initiated based on findings (4), followed by regular follow-up (5). Image created with BioRender.com. https://app.biorender.com/user/ (accessed on 1 January 2026).

The reasons for these delays could be multifactorial. Firstly, the diagnostic journey for patients with IBD often begins with a wide range of nonspecific gastrointestinal (GI) and systemic symptoms [2], which, in the initial stages, may erroneously be attributed to other more common GI conditions (such as irritable bowel syndrome or GI infections) [2]. In some cases, they could even be dismissed as a result of stress or dietary factors, until the time the patient presents repeatedly with persistent or worsening symptoms. Secondly, healthcare professionals, particularly in the community, face challenges in recognising and distinguishing IBD from other GI conditions. The location and type of disease could also impact delayed diagnosis [3]. Thirdly, the lack of specific and easily accessible diagnostic tests such as endoscopic procedures and imaging studies also contributes to the delay. In addition to clinical hurdles, delays in diagnosis can also result from patient-related factors. They may delay seeking medical attention due to fear, embarrassment, or a belief that their symptoms are transient and will probably resolve on their own. Others may face genuine barriers in accessing healthcare services, such as long wait times for appointments or limited access to gastroenterologists. The conventional patient journey within the community with primary care and the process within a hospital setting after referral by a GP are illustrated in Figure 1A,B.

The consequences of a delayed diagnosis of IBD can be far-reaching. Chronic untreated inflammation can lead to complications like intestinal strictures, abscesses, fistulae and significant nutritional compromise [4], thereby increasing the likelihood of patients requiring advanced therapies and/or intestinal surgery early on the disease journey [5,6].

In addition to the physical complications that patients endure, the psychological impact of delayed diagnosis cannot be overlooked. The uncertainty and anxiety experienced by patients during the prolonged diagnostic journey can significantly impact their mental well-being, leading to increased stress, depression, and reduced overall quality of life [7].

Although there is growing evidence that delayed diagnosis has a detrimental effect in both UC and CD [8], many gaps remain in our understanding of the multiple factors that lead to delays. Moreover, these can vary from one hospital/health system to another. A detailed understanding and reporting of both health-system related, and patient-related factors will help identify areas that clinicians may find useful, including bottlenecks.

## 2. Aims of This Study

The time leading up from onset of symptoms to formal diagnosis of IBD is considered a delay if the duration is beyond accepted timelines. Delays are common in the health system due to various factors, and patients are inevitably left untreated.

The aims of this study were to understand the impact of delays in the diagnosis of IBD and report on IBD-related adverse outcomes (AOs). In this retrospective study, we aimed to study the following:

- To record any delays faced by newly diagnosed IBD patients at our centre.
- If so, to understand the various points at which there are delays and identify bottlenecks.
- To understand the factors leading to delays, i.e., patient-related factors, community-related factors, or hospital-related factors.
- To understand if the clinical outcomes were worse in patients who faced a delay compared to those who did not.

## 3. Patients and Methods

All new patients referred by the primary care (community health care) general practitioner (GP) to the gastroenterology services at our tertiary care centre for a suspected diagnosis of IBD over a 5-year period (January 2013 to December 2017) were identified using Electronic Medical Records (EMRs). All relevant demographic and clinical information was collected from the hospital EMRs.

**Figure 2:**
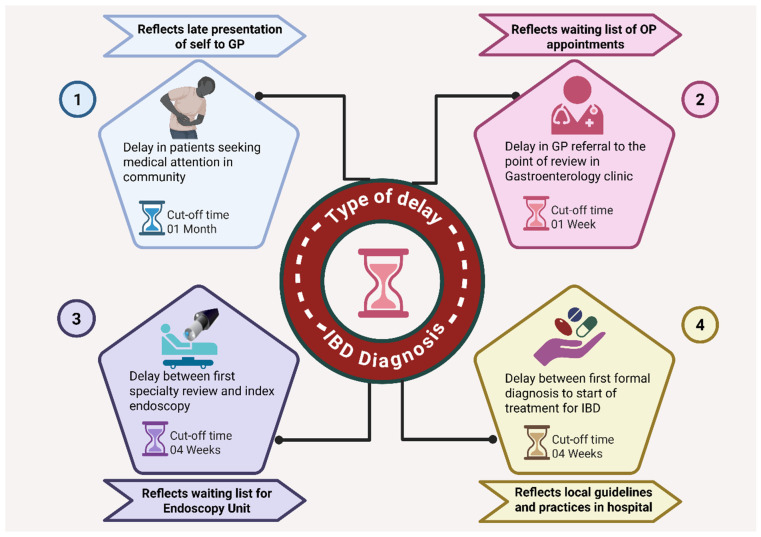
Types of delays contributing to inflammatory bowel disease (IBD) diagnosis. Schematic representation of four key types of diagnostic delays in IBD. 1. Delay in patients seeking medical attention in the community (cut-off: 1 month) reflects late presentation of symptoms to general practitioners (GPs). 2. Delay in GP referral to the point of review in the gastroenterology clinic (cut-off: 1 week) reflects outpatient waiting times. 3. Delay between first speciality review and index endoscopy (cut-off: 4 weeks) reflects waiting list for endoscopy units. 4. Delay between first formal diagnosis and initiation of treatment for IBD (cut-off: 4 weeks) reflects local hospital practices and treatment pathways. Together, these categories highlight patient-, system-, and hospital-level factors influencing the timeliness of IBD diagnosis and management. Image created with BioRender.com https://app.biorender.com/user/ (accessed on 1 January 2026).

For the purposes of this study, different delay types were defined, and separate cut-off times were assigned by the investigators for each delay type. The cut-off times were assigned based on best average hospital waiting times for IBD benchmarking in the National Health Services, UK (NHS) [9]. These benchmarks are from the pre-pandemic period within the NHS. We considered reasonably stringent cut-offs given that our centre is a tertiary care centre with a large, dedicated service for patients with IBD as there is an expectation to manage patients swiftly and efficiently. The definitions of delay type and the details of cut-off times are provided in Table 1 and Figure 2. All primary care referral letters and clinic letters in the hospital were scrutinised for each episode for all included patients. The reasons for delays in patient journey until start of treatment and data on pre-defined AOs (steroid and other rescue therapies, hospitalisation, surgery including perianal, and death) were recorded for each patient until the end of June 2021.

### 3.1. Statistical Analysis

Statistical analyses were performed in R (packages survival and survminer).

Time-to-event analyses were conducted using Cox proportional hazards regression in a counting process (start–stop) form to allow for multiple adverse events per patient. Robust sandwich variance estimates clustered by patient identifier were used to account for within-patient correlation. Hazard ratios (HRs) with 95% confidence intervals (CIs) are reported. Statistical significance was assessed using two-sided Wald tests.

Survival curves for patients with and without diagnosis delay were derived from the fitted Cox model and are displayed with 95% confidence bands.

The association between delay and initiation of biologic therapy during follow-up (binary outcome) was evaluated using univariate logistic regression. Results are presented as odds ratios (ORs) with 95% confidence intervals based on profile likelihood and two-sided Wald *p*-values.

Analyses were conducted using complete case data. A two-sided *p*-value < 0.05 was considered statistically significant.

### 3.2. Ethical Considerations

The study was conducted in accordance with the Declaration of Helsinki, and approved by the Hospital Clinical Governance and Ethics committee (protocol code CARMS-15813 and date of approval 15 July 2019).

## 4. Results

Among a large number of referrals from the GP to the specialist, patients who underwent investigations in our centre and reached a final definitive diagnosis of IBD were first screened. Stringent criteria were applied for patient selection (for e.g., pre-existing diagnosis or new-to-area patients with IBD were excluded) and only newly diagnosed patients with IBD were included.

**Figure 3:**
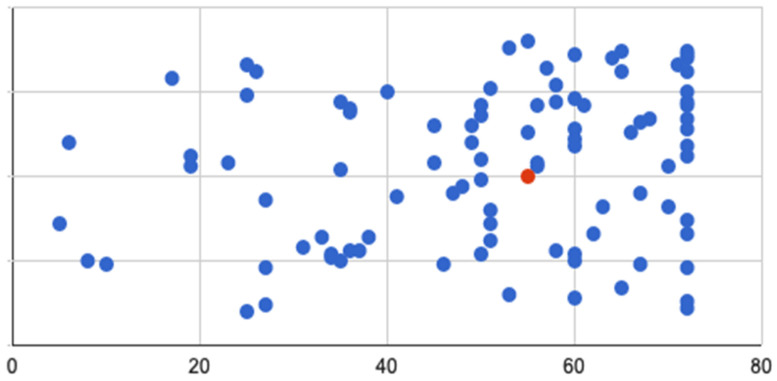
Follow-up data for all patients (in months). In this plot, X-axis indicates time in months. Blue dots indicate patient’s maximum follow-up at specific time periods. Red dot indicates median follow-up for cohort.

A total of 105 patients were identified (M = 58; median age = 32 y), with a median follow-up of 55 months (range 5–72 m). The demographics of patients are provided in Table 2. The most frequent presenting complaints were abdominal pain (44, 41.9%), loose stools (40, 38.1%), bloody diarrhoea (37, 35.2%), and bleeding from the rectum (33, 31.4%), and only 16% declared a family history. A total of 65, 27 and 13 patients had a final diagnosis of ulcerative colitis, Crohn’s disease, and IBD-unclassified, respectively, and were analysed collectively. The breakdown of clinical details are provided in Table 3. The number of patients with follow-up durations are illustrated in a plot graph (Figure 3).

In our total cohort, we identified delays at almost every step. The longest delay types in order were

- Delay in patients seeking medical attention (median = 4 months; range 1 to 84 months);
- Delay between GP referral to gastroenterology clinic appointment (median = 5 weeks; range 1 to 30 weeks);
- Delay between first specialty review and index endoscopy (median = 3 weeks; 1 to 36 weeks);
- Delay between first formBal diagnosis to start of treatment for IBD (median = 2 weeks; range 0 to 12 weeks).

After patient stratification based on these delay types and applying the specific cut-off times for each, we noted a statistically significant difference in survival without AOs for all patients when comparing delayed versus not delayed.

**Figure 4:**
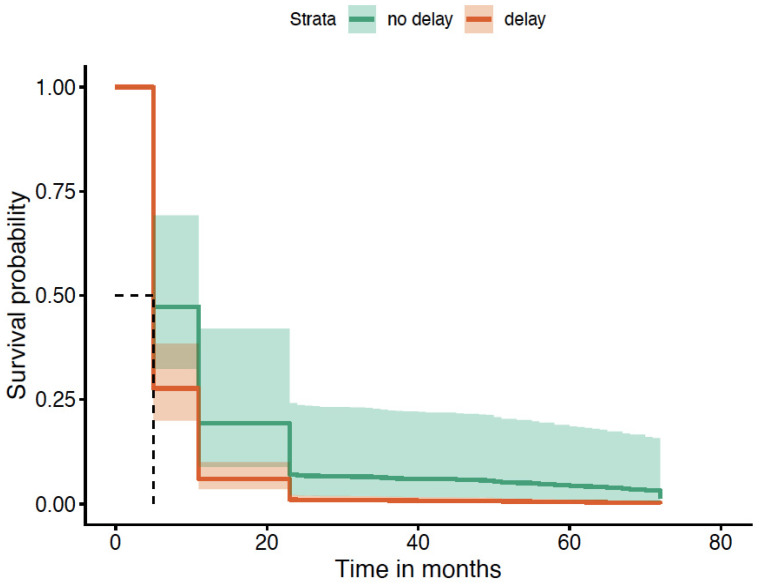
Time to seek medical attention of >1 month (survival without AOs; *p* = 0.004). K-M curve illustrates the higher probability of AOs due to delay, and curves remain separated throughout follow-up period. The dashed lines in the Kaplan–Meier survival plot are marking the median survival time. The horizontal dashed line (~0.5) represents a survival probability of 50%. The vertical dashed line shows the time point at which the survival curve crosses 50%.

When patients delayed seeking medical attention beyond 1 month of symptom onset, they were more likely to suffer AOs up to at least 60 months compared to those who did not delay. This was statistically significant and is illustrated in Figure 4.

**Figure 5:**
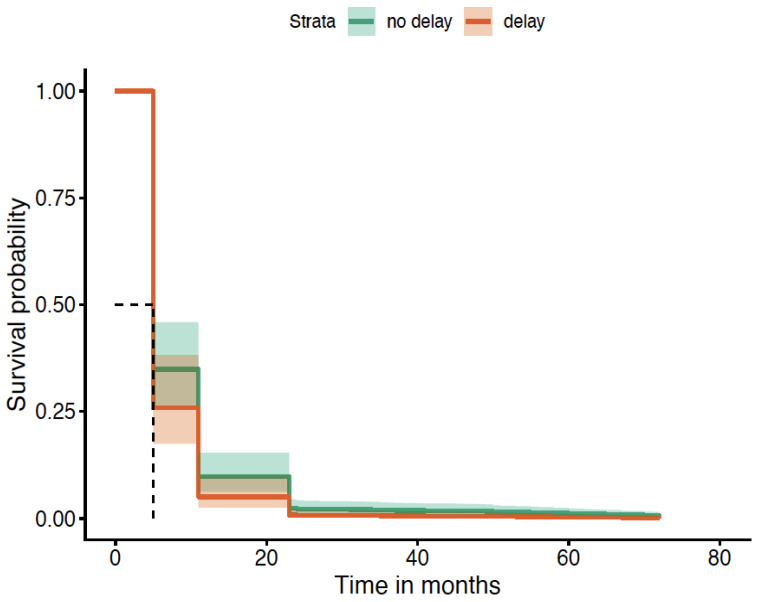
Time from GP referral to specialty review of >1 week (survival without AO; *p* = 0.048). K-M curve illustrates higher probability of AOs due to delay. The dashed lines in the Kaplan–Meier survival plot are marking the median survival time. The horizontal dashed line (~0.5) represents a survival probability of 50%. The vertical dashed line shows the time point at which the survival curve crosses 50%.

When patients faced a delay of more than 1 week between referral from primary care to their first specialty review at the gastroenterology clinic, they were more likely to suffer AOs. This is illustrated in Figure 5.

**Figure 6:**
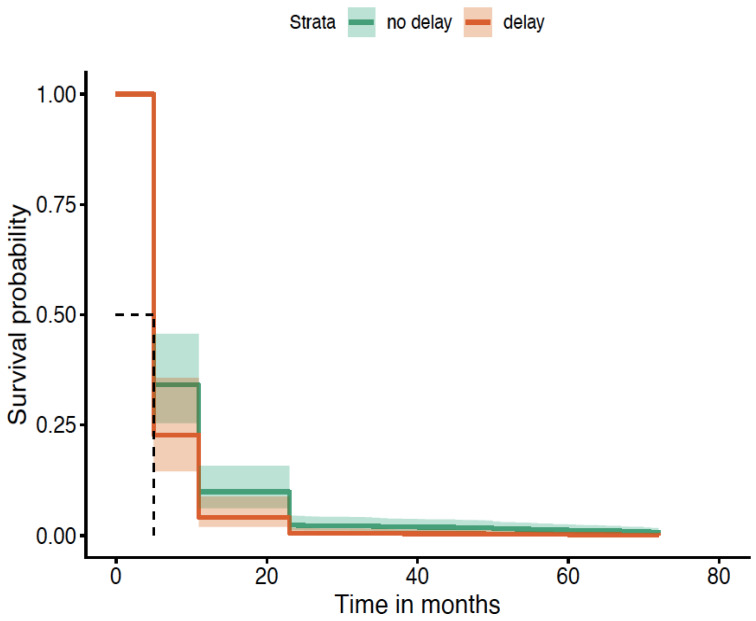
Time from clinic review to index endoscopy of >4 weeks (survival without AOs; *p* = 0.01). K-M curve illustrates higher probability of AO due to delay. The dashed lines in the Kaplan–Meier survival plot are marking the median survival time. The horizontal dashed line (~0.5) represents a survival probability of 50%. The vertical dashed line shows the time point at which the survival curve crosses 50%.

When patients faced a delay of more than 4 weeks within the health system between their first specialty review and index endoscopy, they were more likely to suffer AOs during the follow-up period for up to 60 months (*p* = 0.01). This is illustrated in Figure 6.

**Figure 7:**
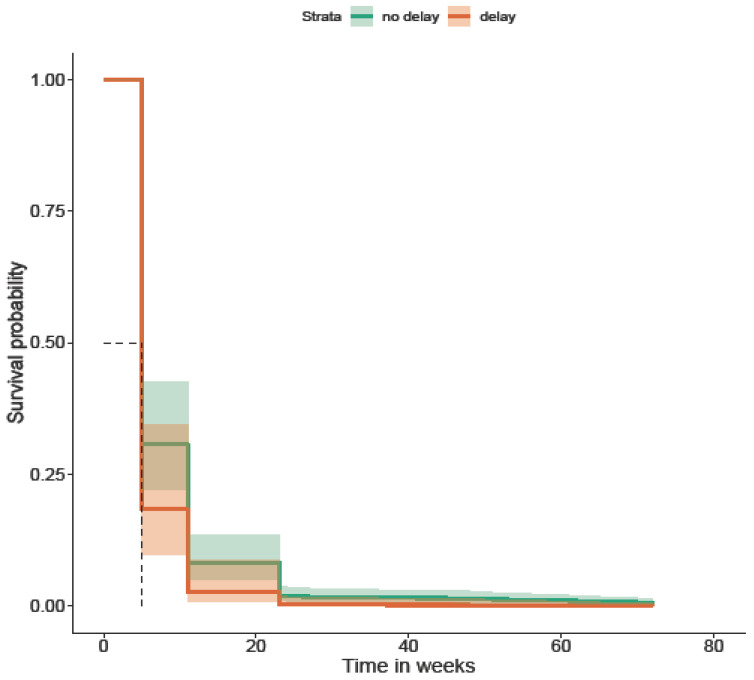
Delay between formal diagnosis and start of treatment (*p* = 0.03). K-M curve illustrates higher probability of AO due to delay. The dashed lines in the Kaplan–Meier survival plot are marking the median survival time. The horizontal dashed line (~0.5) represents a survival probability of 50%. The vertical dashed line shows the time point at which the survival curve crosses 50%.

When patients faced a delay of more than 4 weeks between their first formal diagnosis and start of treatment for IBDs, they were more likely to suffer AOs during the follow-up period for up to 60 months (*p* = 0.03). This is illustrated in Figure 7.

### Delays and Biologic Use During Disease Course

We also examined if delays in diagnosis influenced the use of biologics during the follow-up period in our cohort. Using “biologic-use” at any time during follow-up as an outcome variable, different delay types were compared, keeping the cut-off times the same as before. It was noted that any delay increased the risk of biologic use during the follow-up period. The details of delay type and odds ratio of biologic use are given in Table 4.

We then combined more than one type of delay to examine if a cumulative effect was able to predict use of biologics. In our cohort, a model that combined delay in seeking medical attention and delay between specialty review to index endoscopy exhibited the best predictive power, with an average AUROC of 0.67.

**Figure 8:**
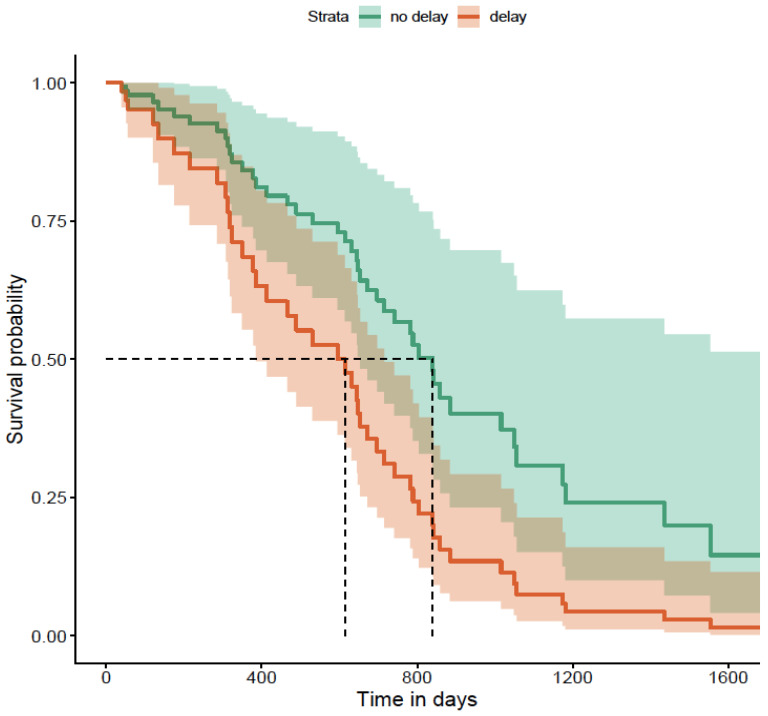
Patient-related delay of >2 weeks and risk of needing biologics (*p* = 0.042). The dashed lines in the Kaplan–Meier survival plot are marking the median survival time. The horizontal dashed line (~0.5) represents a survival probability of 50%. The vertical dashed line shows the time point at which the survival curve crosses 50%.

Upon further analysis of patient-related and health-system-related delays, there was a statistically significant increase noted in the risk of requiring biologics at any time during the follow-up period. The risk of requiring biologics was statistically significantly increased with a patient-related delay of more than 2 weeks compared less than 2 weeks. Figure 8 illustrates this risk, and it is noted that this remained higher for more than 4 consecutive years.

**Figure 9:**
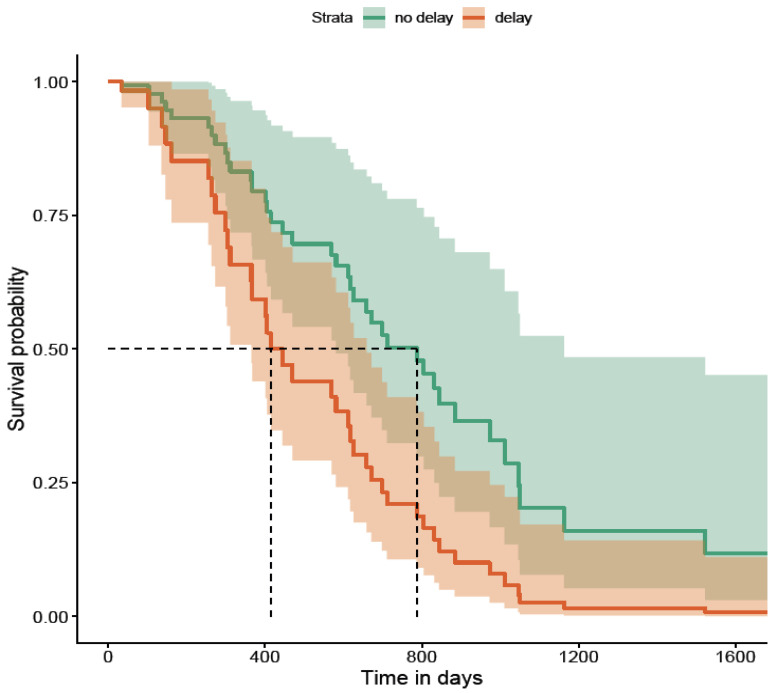
Health-system-related delay of >6 weeks and risk of needing biologics (*p* = 0.021). The dashed lines in the Kaplan–Meier survival plot are marking the median survival time. The horizontal dashed line (~0.5) represents a survival probability of 50%. The vertical dashed line shows the time point at which the survival curve crosses 50%.

Once patients were within the health system, a cumulative delay of more than 6 weeks increased the risk of biologic therapy over the next 4 years. This is illustrated in Figure 9.

## 5. Discussion

Delays in diagnosis are quite common in most health systems. Timely diagnosis is more likely to have a favourable outcome. However, there may be many reasons why timely diagnosis is not always achieved. The poor outcomes due to delayed diagnosis have wide-ranging effects. There have been multiple studies looking into this aspect, and most have focused on the median delays per disease type and their outcomes. Our focus in this study was to understand if there is a period beyond which the delay becomes detrimental. This aspect is particularly important and pertinent now given the significant burdens many health systems are facing during the post-pandemic recovery period. It is important to understand the factors leading to delays to rectify the situation and offer solutions.

In one study, Vavricka et al. reported the risk factors that led to delays in the diagnosis of IBDs. The authors used data from a comprehensive Swiss national IBD database during a three-year period. They found that the median delay in patients with CD from first onset of IBD-related symptoms to IBD diagnosis was 9 months, significantly longer than the of patients with UC, which was 4 months. We found no significant differences between UC and CD. They also reported that younger patients with CD and ileal disease had the highest risk of long diagnostic delays, probably due to an overlap of IBS-type symptoms [3]. In another study, the authors reported a similar risk for patients with CD. In addition, active smoking status and symptom onset during summer were found to be independent risk factors. A long diagnostic delay was noted to increase the risk of stricturing disease (OR, 3.38; *p* < 0.01), fistulae (OR, 2.64; *p* = 0.08), and IBD-related surgery [10].

In our cohort, for each of the delay types defined, there were generally more patients who faced a delay than those who did not. We were able to compare these within each delay type.

### 5.1. Delay in Patients Seeking Medical Attention

The delay of this patient-related delay type was the longest, with a median of 4 months. This is consistent with the findings of other studies [8]. Although there are prospective studies that have reported a significantly longer median time to diagnosis for CD [11], this was not noted in our cohort. For this delay type, when patients waited beyond a month to seek medical attention, they were more likely to have AOs, and they were more likely to need biologics to manage their condition. In real-world settings, patients do not always consult the GP immediately for various reasons. Moreover, it remains unclear if a particular time (to wait) is acceptable. Based on our findings, it would be reasonable to educate patients not to wait beyond 2–3 weeks to seek medical attention.

### 5.2. Delay Between GP Referral to Gastroenterology Clinic Appointment

This delay type is health-system-related, with a median time of 5 weeks in our cohort. In our cohort, a delay of more than 1 week resulted in worse outcomes. This was perhaps the most likely bottleneck that affected hospitals, which was particularly worse after the COVID-19 pandemic. There are no reliable data on the breakdown of delay types comparing clinical outcomes. Most studies report a cumulative delay only. This particular delay reflects the waiting lists t hospitals before a patient is offered an appointment at the specialty clinic. The waiting time for this critical stage in the patient journey has likely increased in almost all healthcare settings due to increasing demand, clearance of the backlog of appointments, lack of workforce, and shortage of experts. As IBDs are complex diseases, it is unlikely that treatment is started empirically in primary care before a formal diagnosis can be established. Also, GPs are more comfortable maintaining treatment as advised by experts than initiating treatment [12,13]. This delay in starting treatment leaves patients in a state of sustained chronic inflammation, thereby worsening outcomes.

### 5.3. Delay Between First Specialty Review and Index Endoscopy

This delay type is the next step in establishing a diagnosis, with a median delay of 3 weeks in our cohort. This reflects the waiting list for endoscopy departments. Endoscopy is an essential tool not only for diagnosis but also for the ongoing assessment of disease activity and the monitoring of patients on advanced therapies. Although the median time was less than the assigned cut-off in our cohort, patients who faced delays beyond 4 weeks had a statistically higher risk of having AOs during follow-up. This was made worse by the backlogs during the pandemic and the post-pandemic recovery. As predicted by experts during the pandemic, patients are waiting longer to undergo procedures [14]. The ever-increasing waiting lists will leave a larger number of patients at risk of having untreated CI and more AOs.

### 5.4. Delay Between First Formal Diagnosis to Start of Treatment for IBDs

This delay type generally varies from one hospital to another based on local policies and guidelines. In our cohort, the median time to start of treatment was 2 weeks, which was better than our cut-off of 4 weeks. Among patients who faced a delay beyond 4 weeks, there was a statistically significantly higher risk of AOs. This delay should ideally be minimal as treatment can be started immediately at the time of contact with patients during their index endoscopy. One study reported more than 75% patients started treatment within 2 weeks of diagnosis [9]. It is possible this proportion may have reduced owing to pressures on outpatient departments during the post-pandemic era. There have been reports of a drop in prescription rates during the immediate post-pandemic phase. One study reported a sharp fall in prescriptions for thiopurines (−81%), with lesser reductions for biologics (−38%) and oral prednisolone (−20%) [15]. This is likely to have improved to some extent but not to baseline.

It is important to consider cumulative delays within the health system of more than 6 weeks, which significantly increase the risk of patients requiring biologics over the next 4 years.

It is clear from our data that any delay in diagnosis, whether patient-related or health-system-related, results in worse outcomes. Our study is one of the few that has analysed and reported the details of the breakdown of delay types and the relevant timelines for each. By doing so, it is possible to focus on those areas identified as causing the longest delays. Such bottlenecks, once identified, can frequently be managed with minor changes in patient flow, referral systems, screening processes, and so on. For example, one of the simplest to implement that could reduce delays in starting treatment would be to provide prescription in endoscopy for patients newly diagnosed with IBDs. Individual centres will need to work on their own bottlenecks, using these timelines as targets to be achieved. Timely diagnosis will very likely result in better outcomes and fewer AOs, ultimately reducing the burden on the healthcare system. Overall, achieving adequate control of chronic inflammation would improve short- and long-term outcomes for patients with IBDs.

The correlation between delay and use of biologic therapy is a complex one to explore. There are various factors that influence the use of biologics, and delayed diagnosis is perhaps one of them. Although it is difficult to quantify the weight of each factor, any delay would mean longer sustained chronic inflammation, leading to complications. This must be interpreted based on the context.

Further subgroup analysis is provided in the Supplementary Material, Table S1.

### 5.5. Strengths and Limitations of This Study

Our study has several strengths. We applied stringent criteria for inclusion to ensure only inception patients were part of the study cohort. This was chosen to ensure that we were able to study the outcomes of sustained chronic inflammation at the start of the disease process. We analysed delay types in great detail and found that specific timelines are relevant to clinical outcomes. We assigned stringent but fair cut-off times to measure the performance of the service, making it robust.

Our study has several limitations. As this was a retrospective study, there were some data missing, particularly data such as smoking status and up-to -ate biomarkers, and it is possible that AOs are under-reported. There is no universally agreed definition of delay in IBDs, which creates a challenge. Our own interpretation of delay is not flawless, and lag intervals could have been misclassified. However, this is characteristic of most real-world studies that are exploring areas with limited evidence. The data was from a single tertiary care centre, and the number of patients included in this study were relatively low. As our centre is a tertiary care centre with a dedicated IBD service, we assumed our timelines may be better than other centres. Despite this advantage, even short delays appear to have an impact on clinical outcomes. Due to the smaller number of patients, we were unable to perform statistically meaningful univariate or multivariate regression analysis to identify risk factors that could predict delays. This may also have introduced some residual confounding, limiting the robustness with which causal interpretation can be made. The data on biologic use must be interpreted with caution due to the smaller numbers in this cohort.

## 6. Conclusions

To conclude, we found from our data that delays, and subsequent untreated chronic inflammation, lead to poor outcomes in patients with newly diagnosed IBD.

Our study found that delays in diagnosis could be detrimental. Whether they are patient-related or health-system-related, they lead to poorer outcomes. There are often several bottlenecks in a service that contribute to delays, increasing AOs in IBD over the follow-up period.

In our cohort, a delay as short as a week between GP referral and specialty review was statistically significant in determining Aos; hence, their negative impact should not be underestimated.

Each delay type is unique and highlights a different area for improvement. Any delay by patients in seeking medical attention from their GP shows that patients may not be aware of the potential seriousness of their GI symptoms. This could be addressed by undertaking patient education and awareness programs locally, regionally, and nationally. Delays in clinical appointments and endoscopy appointments can vary based on the centre. This particular problem is pertinent during the post-pandemic recovery period as almost every hospital in the UK has been struggling to clear backlogs, while simultaneously grappling with unprecedented demands placed on an already stretched workforce. This is also applicable to specialist IBD centres, where our data is likely to have implications for service delivery and planning. The data on the high proportion of AOs may prove supportive in planning business cases to redesign services, add workforce and prioritise IBD patients, to minimise the damage caused by excessive delays. Our data also suggests that cumulative delays in the hospital increase the use of biologics in consecutive years. This will add to pressure on IBD teams and infusion suites at hospitals. Specialist services will need to allocate resources appropriately to meet this demand in the long term. Both hospitals and policy makers will need to consider this and allocate the necessary resources to minimise delays and meet the standards of best practice.

## Figures and Tables

**Table 1:** Definition of delay type and cut-off times for each type.

Type of Delay	Cut-Off Time
Delay in patients seeking medical attention in community (reflects late self-presentation to GP)	1 month
Delay in GP referral to the point of review in gastroenterology clinic (reflects waiting list for outpatient appointments in hospital)	1 week
Delay between first specialty review and index endoscopy (reflects waiting list for endoscopy)	4 weeks
Delay between first formal diagnosis to start of treatment for IBD (reflects local guidelines and practices in hospital)	4 weeks

**Table 2:** Demographics and classification.

Age & Sex	N = 105
Median age	32 years (range 21–82 years)
Sex	Male = 58; Female = 47
**Race**	
Caucasian	65 (62%)
Asian	28 (27%)
Afro-Caribbean	1 (1%)
Unknown/Unreported	11 (10%)
**Disease type at first diagnosis**	
Ulcerative colitis	65 (62%)
Crohn’s disease	27 (26%)
IBD-unclassified	13 (12%)
**Montreal classification for CD**	
Age	
A1	2
A2	22
A3	3
Location	
L1	7
L2	6
L3	13
L4	0
L3 + L4	1
Behaviour	
B1	21
B2	3
B3	0
B1 + perianal	3
**Ulcerative colitis extent**	
E1	12
E2	36
E3	17

**Table 3:** Clinical data—symptoms, medications at start, and follow-up period.

Symptoms at Presentation	Number of Patients
Abdominal pain	44
Loose stools	40
Loose stools with blood in stools	37
Bleeding from rectum	33
Weight loss	16
Vomiting	5
**Medications started at first diagnosis**	
Oral 5-ASA	48
Oral Steroids	33
Rectal 5-ASA	32
Rectal steroids	8
5-ASA and steroids	6
Thiopurines	5
Biologics	5
**Details of follow-up**	
Median follow-up period of cohort	55 months (range 5–72 months)
Patients with first follow-up <6 months	101
Patients with first follow-up 6–12 months	2
Patients with first follow-up 12–24 months	2
Patients with first follow-up >24 months	0

**Table 4:** Risk of biologic use due to delays in diagnosis.

Type of Delay	Risk of Biologic Use During Follow-Up (OR with CI)
Delay in patients seeking medical attention in community	1.08 (1.03, 1.17)
Delay between GP referral to point of review at gastroenterology clinic	1.10 (1.0, 1.23)
Delay between first specialist review and index endoscopy	1.09 (1.01, 1.21)
Delay between first formal diagnosis to start of treatment for IBDs	1.08 (1.0, 1.21)

## Data Availability

The original contributions presented in this study are included in the article/Supplementary Material.

## Supplementary Materials

The following supporting information can be downloaded at: https://www.mdpi.com/article/10.3390/diagnostics16071043/s1, Figure S1: Impact of delay from GP referral to clinic review on outcomes in ulcerative colitis, Figure S2: Impact of delay in seeking medical attention on outcomes in ulcerative colitis, Figure S3: Impact of delay to endoscopy on outcomes in ulcerative colitis; Table S1: Frequency of individual adverse outcomes during follow-up.
