# Supplementary material for: Impact of Delayed Diagnosis in IBD on Clinical Outcomes and Healthcare Delivery
Source: Diagnostics (Basel). 2026 Mar 30;16(7):1043. doi: 10.3390/diagnostics16071043 (PMC13074209; doi:10.3390/diagnostics16071043)
Supplement: Supplementary file 1 [file diagnostics-16-01043-s001.zip › diagnostics-4131633-supplementary.pdf]

Supplementary Material -A

This table summarises the frequency and proportion of predefined adverse outcomes (AOs) observed during follow-up in patients with newly diagnosed inflammatory bowel disease (IBD) included in the study cohort (N = 105). Outcomes recorded from diagnosis until the end of follow-up (June 2021) included steroid therapy (topical, oral, or intravenous), rescue therapy (biologics or cyclosporine), hospitalisation, IBD-related surgery (Crohn's disease or ulcerative colitis), and death. Major adverse outcomes comprised hospitalisation, surgery, and death, while steroid and rescue therapies were classified as minor adverse outcomes. Patients could experience more than one outcome; therefore, categories are not mutually exclusive. Percentages are calculated using the total cohort (N = 105).

Supplementary Table S1. Frequency of individual adverse outcomes during follow-up

Adverse outcome	Number of patients (n)	Proportion of cohort (%)
Topical steroids	85	81.0
Oral steroids / steroid therapy	—	—
Intravenous steroids	37	35.2
Hospitalisation related to IBD	62	59.0
Rescue therapy with biologics	47	44.8
Rescue therapy with cyclosporine	1	1.0
Surgery for Crohn's disease	16	15.2
Surgery for ulcerative colitis	11	10.5
Death	0	0

Supplementary Material – B

This sections provides details of Cox proportional hazard analysis on UC patients.

Delay from GP to clinic review >6 weeks, UC only (p=0.0.034)

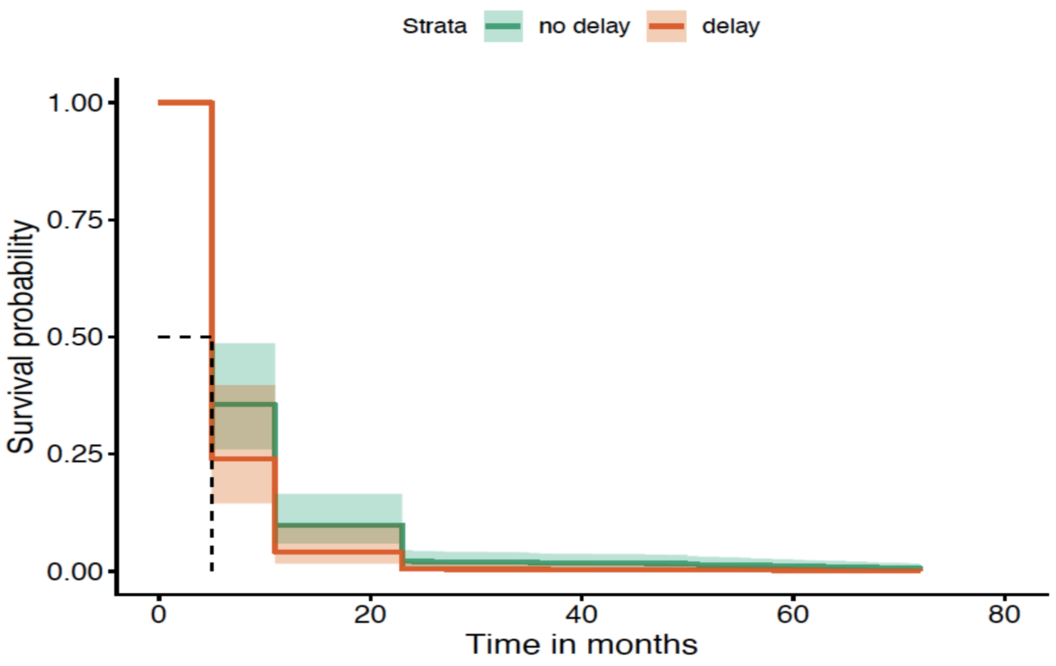

Supplementary Figure 1. Impact of delay from GP referral to clinic review on outcomes in ulcerative colitis. Kaplan–Meier curves showing survival probability over time in patients with **ulcerative colitis (UC)** stratified by time from general practitioner (GP) referral to specialist clinic review: **no delay (≤6 weeks, green)** versus **delay (>6 weeks, orange)**. Shaded areas represent 95% confidence intervals. Patients experiencing a delay of more than 6 weeks demonstrated significantly poorer survival compared with those reviewed within 6 weeks (**log-rank p = 0.034**). The dashed lines indicate the approximate time point at which survival probability drops below 50%.

Delay to seeking medical attention > 1 month UC only (p=0.0.0004)

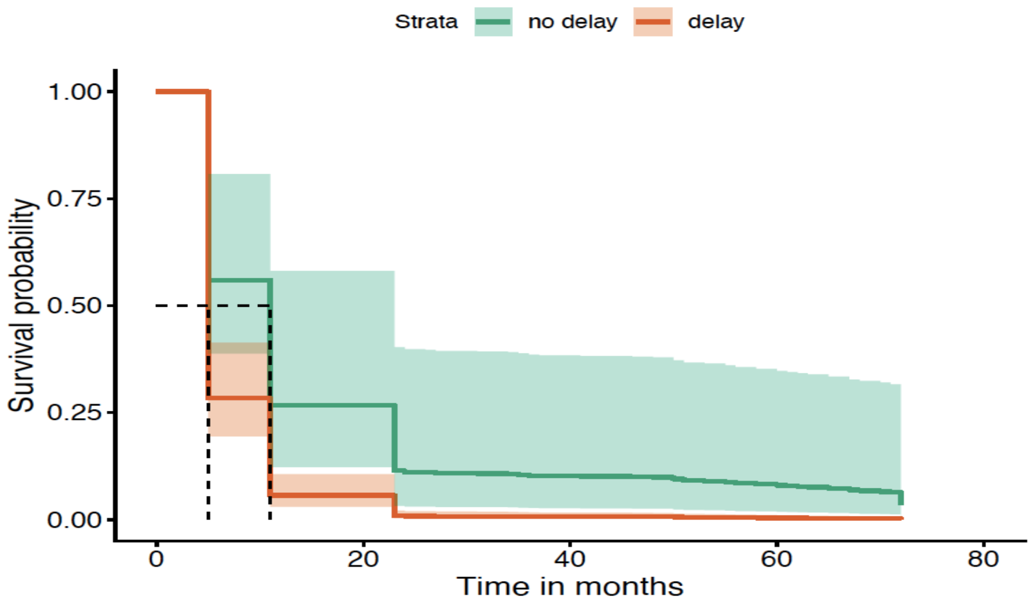

Supplementary Figure 2. Impact of delay in seeking medical attention on outcomes in ulcerative colitis. Kaplan–Meier curves showing survival probability over time in patients with ulcerative colitis (UC) stratified by time from symptom onset to seeking medical attention: no delay (≤1 month, green) versus delay (>1 month, orange). Shaded areas represent 95% confidence intervals. Patients who delayed seeking medical care for more than one month had significantly poorer survival compared with those who sought care within one month (log-rank $p = 0.0004$). Dashed lines indicate the approximate time point at which survival probability falls below 50%.

Delay to endoscopy >4 weeks UC only (p=0.021)

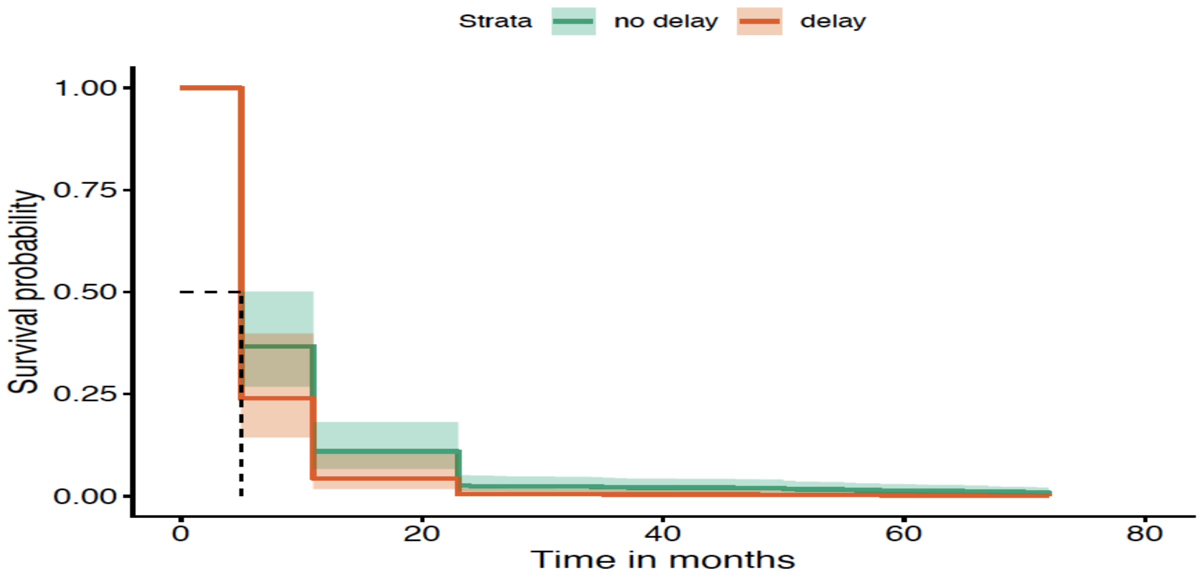

Supplementary Figure 3. Impact of delay to endoscopy on outcomes in ulcerative colitis. Kaplan–Meier curves showing survival probability over time in patients with **ulcerative colitis (UC)** stratified by time from initial presentation to diagnostic endoscopy: **no delay (≤4 weeks, green)** versus **delay (>4 weeks, orange)**. Shaded areas represent **95% confidence intervals**. A delay of more than four weeks to endoscopic evaluation was associated with significantly poorer survival compared with patients undergoing endoscopy within four weeks (**log-rank p = 0.021**). Dashed lines indicate the approximate time point at which survival probability falls below 50%.
